# Reciprocating Arc Silicon Strain Gauges

**DOI:** 10.3390/s23031381

**Published:** 2023-01-26

**Authors:** Ji-Hoon Han, Sung Joon Min, Joon Hyub Kim, Nam Ki Min

**Affiliations:** 1 KIURI Center for Hydrogen Based Next Generation Mechanical System, Inha University, Incheon 21999, Republic of Korea; 2 Department of Stretchable Task Team, LG Display, Seoul 07796, Republic of Korea; 3 Department of Nanomechatronics Engineering, Pusan National University, Busan 46241, Republic of Korea; 4 Department of Electro-Mechanical Systems Engineering, Korea University, Sejong 30019, Republic of Korea

**Keywords:** reciprocating arc strain gauge, bulk micromachining, glass frit bonding, pressure sensor, steel diaphragm

## Abstract

Currently, silicon-strain-gauge-based diaphragm pressure sensors use four single-gauge chips for high-output sensitivity. However, the four-single-gauge configuration increases the number of glass frit bonds and the number of aluminum wire bonds, reducing the long-term stability, reliability, and yield of the diaphragm pressure sensor. In this study, a new design of general-purpose silicon strain gauges was developed to improve the sensor output voltage while reducing the number of bonds. The new gauges consist grid patterns with a reciprocating arc of silicon piezoresistors on a thin glass backing. The gauges make handling easier in the bonding process due to the use of thin glass for the gauge backing. The pressure sensors were tested under pressure ranging from 0 to 50 bar at five different temperatures, with a linear output with a typical sensitivity of approximately 16 mV/V/bar and an offset shift of –6 mV to 2 mV. The new approach also opens the possibility to extend arc strain gauges to half-bridge and full-bridge configurations to further reduce the number of glass frit and Al wire bonds in the diaphragm pressure sensor.

## 1. Introduction

Diaphragm-type pressure sensors are widely used to measure high pressure in most industrial process control systems, the automotive industry, and medical science [1,2,3,4,5]. The most popular pressure-sensing diaphragm used in pressure sensors is a circular stainless-steel plate fixed around the edge and exposed to the pressure medium on one side [6]. When the pressure to be measured is applied through the medium, the diaphragm deflects to an extent proportional to the magnitude of the pressure. This displacement is measured by four strain gauges arranged in a bridge circuit configuration. The output voltage from the bridge is a function of the resistance change due to the strain in the diaphragm. This arrangement automatically provides compensation for environmental temperature changes.

**Figure 1:**
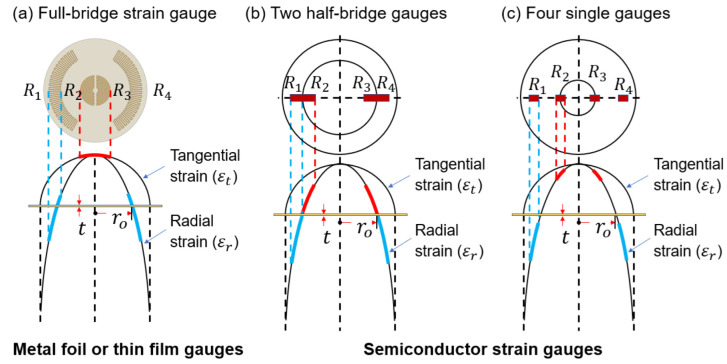
Examples of positions of strain gauges on a pressure-sensing circular diaphragm: (**a**) full-bridge metal foil or thin film gauges; (**b**) two silicon half-bridge gauges; and (**c**) four single silicon gauges.

The stress exerted on the diaphragm can be either tensile or compressive; hence, the location of strain gauges is important. Figure 1 shows the strain distribution and gauge positions in a rigidly clamped circular diaphragm under uniformly applied pressure [7]. The radial and tangential strains have identical maximum tensile strains at the center of the diaphragm. However, as the radius increases, the tangential strain becomes zero at the periphery, whereas the radial strain decreases rapidly, becoming compressive and equal to twice the center strain at the edge. Diaphragm-type pressure transducers typically use two different strain gauges: metallic strain gauges and semiconductor strain gauges. Four specially designed strain gauges are included in the proposed system, two of which are used to measure the tensile stress (positive strain), with the other two gauges measuring the compressive stress (negative strain). Figure 1 shows examples of the arrangement of four metallic and silicon strain gauges. Metallic gauges have a low gauge factor (typically around 2), which indicates that the low output from the gauge bridge circuit has to be amplified by a high-gain amplifier. The metallic strain gauges are designed in the traditional circular pattern [8,9,10,11], allowing the incorporation of a full bridge into a single strain gauge, as shown in Figure 1a, to take advantage of the maximum tangential and radial strains described above. Gauges R_1_ and R_4_, which are placed near the periphery, respond to compressive radial strain, respectively, whereas gauges R_2_ and R_3_ placed around the center respond to tensile tangential strain, respectively.

The development of semiconductor (piezoresistive) strain gauges provides a solution to the low-output problem of metal gauges, as they have a sensitivity up to 100 times greater than that of metallic gauges due to the piezoresistive effect [12,13]. Commercially available silicon strain gauges for diaphragm pressure sensors are designed with a single stain gauge [14,15] or half-bridge configurations [16,17,18,19,20,21], as shown in Figure 1b,c. The half-bridge chip consists of two strain gauges and is very popular for commercial usage because of a reduced number of bonds to the steel diaphragm and faster installation. However, it suffers from low output voltage because it is located in the low strain region of the diaphragm. The main advantage of using a single gauge with a much larger output is that it is attached to the maximum strain area of the diaphragm, in contrast to the half-bridge chip. When the number of glass frit bonds and aluminum (Al)–wire bonds increases, the long-term stability, reliability, and yield of strain-gauge-based pressure sensors are affected.

In this work, the design methodology, simulation, and fabrication of reciprocating arc strain gauges based on piezoresistance in silicon are discussed. These gauges were placed in the high strain region of the diaphragm in a Wheatstone bridge configuration and function in the same manner as a circular metal gauge (Figure 1a) but with higher differences in total gauge output. Reciprocating arc silicon (Si) strain gauge-type sensors offer a wide variety of features, such as an easy bonding process due to the reduced number of gauge dies compared to the four-single-gauge configuration shown in Figure 1c, high-precision measurements with excellent linearity and consistency, and much higher output voltage than the Si half bridges shown in Figure 1b. The units used in this study are based on Si units except for pressure, which uses a CGS unit bar for notational convenience.

## 2. Sensor Design and Simulation

**Figure 2:**
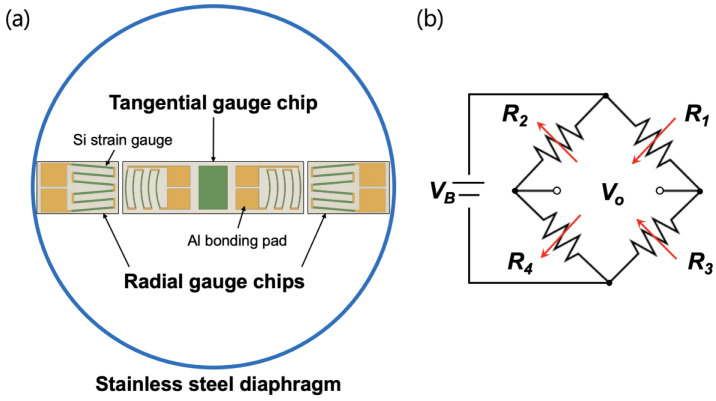
(**a**) Top view of the arrangement of silicon strain gauges on a pressure-sensing circular diaphragm. (**b**) Composition of a Wheatstone bridge circuit applied to a silicon strain gauge.

Figure 2a shows the pressure sensor design with reciprocating arc silicon strain gauges. Usually, the steel diaphragm in a pressure sensor is modified into a circular shape with all the edges clamped. The silicon strain gauges were placed on the surface of the diaphragm and were then connected in a balanced Wheatstone bridge configuration using aluminum wire bonding, as shown in Figure 2b. To achieve a differential signal voltage from pressure changes, the four gauges were oriented such that the resistance change in each gauge was equal in magnitude but opposite in sign. The tangential gauge chip around the center consisted of two reciprocating arc-shaped strain gauges measuring the tangential (positive) stress. The two single gauges near the periphery were in the form of a meander with six linear piezoresistors connected in series that responded to compressive (negative) strain.

**Figure 3:**
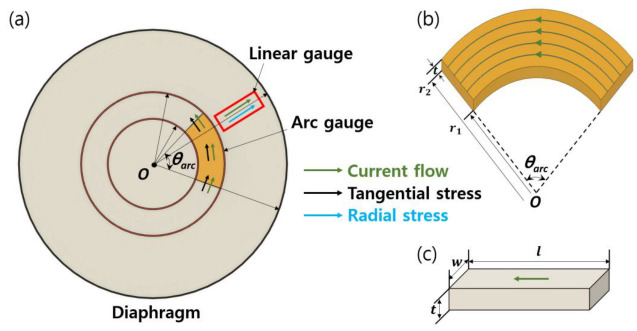
Design principle of arc and linear gauges on a circular diaphragm. (**a**) Locations of tangential (arc) gauge and radial (linear) gauge. Models of (**b**) arc-shaped and (**c**) linear piezoresistors.

Figure 3a shows the design principle of an arc strain gauge responding to tangential strain. The gauge current was in the circumferential direction to measure a tangential component of strain. The unstressed resistance of the arc geometry (Figure 3b) was determined by solving Laplace’s equation [22] in cylindrical coordinates. Assuming uniform current and electric field and doping concentration within $\theta_{arc}$, for simplicity, the solution to Laplace’s equation is expressed as follows for an arc-shaped piezoresistor

(1) $$R_{arc}=R_{s}\frac{\theta_{arc}}{\ln\left(r_{2}/r_{1}\right)}$$

where $R_{s}=\frac{\rho }{t}$ is the sheet resistance, and ρ and t are the resistivity and thickness of silicon layer, respectively.

**Figure 4:**
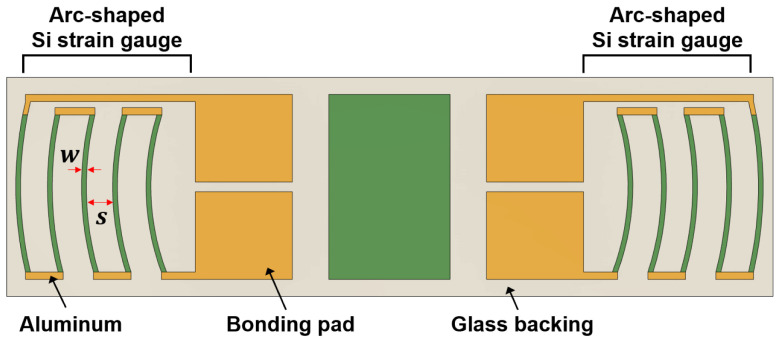
A newly designed tangential strain gauge chip and its design parameters.

Arc-shaped piezoresistors can be configured with different numbers of turns depending on the desired gauge resistance and sensitivity. In this paper, a five-turn configuration was selected, as shown in Figure 4. Therefore, the total resistance of tangential strain gauge can be expressed as

(2) $$R_{tangential\;}=5R_{S}\frac{\theta_{arc}}{ln\left(r_{2}/r_{1}\right)}$$

In Equation (2), the factor of 5 was selected because five identical arc piezoresistors were connected by a turn-around aluminum loop in order to achieve tangential current flow through strain gauge and to minimize the transverse sensitivity of a measuring piezoresistor [23]. The gauge chips measuring the tangential strain were designed in dual-strain gauge patterns, as shown in Figure 4, which has both gauges on a common glass backing like conventional silicon half-bridge chips [20,21]. Compared to the single strain gauges shown in Figure 1c, this design offers benefits such as easier and faster installation and alignment.

**Figure 5:**
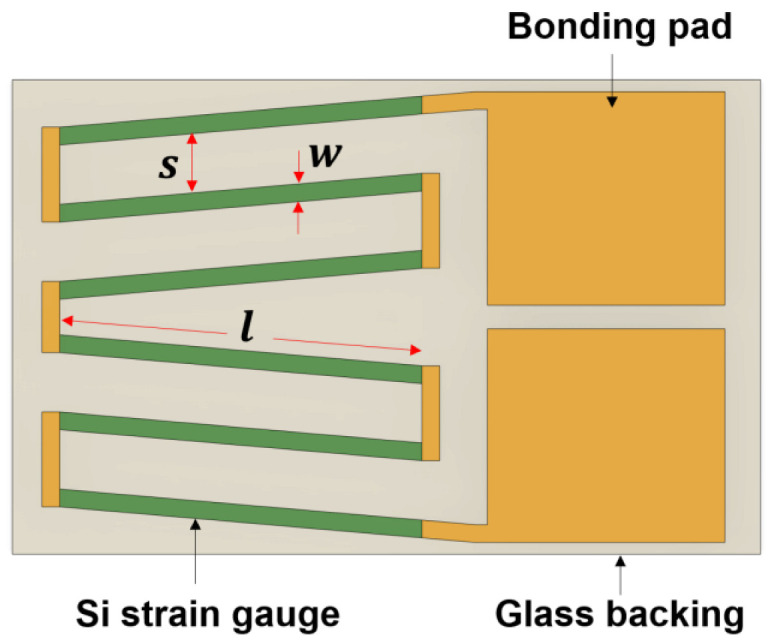
Single strain gauge pattern designed for radial strain sensing and its design parameters.

For a linear strain gauge, the current is in the same direction as the piezoresistor orientation, as shown in Figure 3c. Therefore, the unstressed resistance of a linear piezoresistor is simply expressed as

(3) $$R_{linear\;}=R_{S}\frac{w}{l}$$

where *w* and *l* are the width and length of the linear resistor, respectively. Figure 5 illustrates the layout of the radial strain gauge, which has six linear piezoresistors connected in series by a short aluminum loop. The electrical resistance of a radial gauge is as expressed as follows

(4) $$R_{radial}=6R_{S}\frac{w}{l}$$

**Figure 6:**
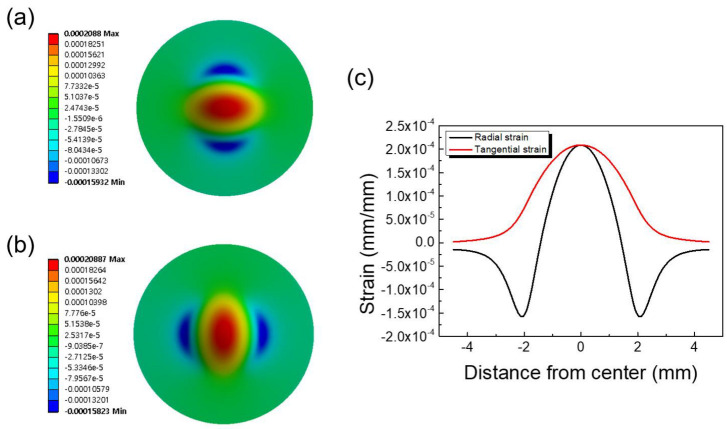
Strain distribution on the surface of a stainless-steel circular diaphragm. Color maps of (**a**) tangential and (**b**) radial strains. (**c**) Two-dimensional plot of tangential and radial strains.

To determine the gauge locations on the top surface of the circular diaphragm, the strain distribution in the pressure-loaded steel diaphragm was obtained from finite element analysis (FEA) by ANSYS, as shown in Figure 6. The material of the diaphragm was 630 stainless steel (SUS 630), which is currently the most widely used material for pressure sensors. The pressure range was determined by the structural parameters of the diaphragm, such as thickness (*h*), radius (*R*), and fillet radius (*r*). The parameters used in the simulation and calculation are as follows: Young’s modulus, *E* = 1.93 × 1011 Pa; Poisson’s ratio, ν = 0.31; available radius, *R* = 3.4 mm; thickness, *h* = 0.4 mm; fillet radius, *r* = 0.5 mm; and applied pressure, *p* = 50 bar.

Figure 6a,b are the strain maps for the tangential and radial directions, respectively. Figure 6c shows the detailed strain distribution along the diameter of the diaphragm. As shown in Figure 6c, both the tangential and radial strains are symmetrical, and an identical maximum value is reached at the center of the circular diaphragm. The tangential strain was always positive and gradually decreased to zero at the periphery of the diaphragm. As the radius increased, the radial strain decreased more rapidly from maximum to zero, becoming negative at the edge due to the presence of the fillet radius. It was clearly observed that in the positive (tensile) strain region, the tangential strain was much larger than the radial component. Therefore, the reciprocating arc gauges were designed to respond to tangential strain. The output voltage of the full-bridge strain gauge circuit was dependent on the average strain under the gauge grids [8,24]. The strain values shown in Figure 6c were used to calculate the average strain experienced by each strain gauge.

## 3. Strain Gauge Fabrication and Bonding

**Figure 7:**
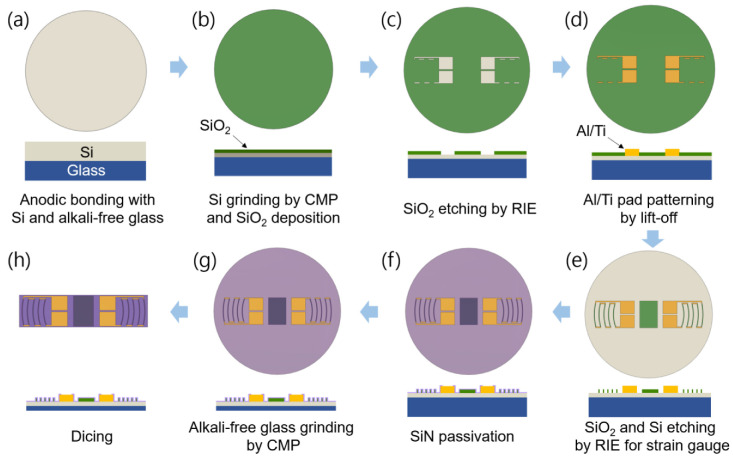
Fabrication process for reciprocating arc-shaped silicon strain gauges.

Figure 7 shows the fabrication procedure for the newly designed arc-shaped and linear gauges. The wafers used in manufacturing were made by anodic bonding a low-resistance 8″ p-type Si wafer and an alkali-free glass wafer at 500 °C and 1750 V for 30 min (Figure 7a). Chemical mechanical polishing (CMP) was used to polish the top silicon of the Si/glass wafer on which the strain gauge was formed to a thickness of around 10 μm. Next, a 50 nm thick silicon dioxide (SiO_2_) sample was deposited on the thinned Si surface using plasma-enhanced chemical vapor deposition (PECVD) (Figure 7b); then, photolithography was performed using a positive photoresist to open a window for the Al pads and the short Al loops at the end of the piezoresistors (Figure 7c). Subsequently, 50 nm thick titanium (Ti) and 800 nm thick Al were successively deposited and patterned by a liftoff process to form the bonding pads and the short end loops, as shown in Figure 7d. A process of depositing Ti as a barrier metal was employed to prevent the destruction of the Al layer in the contact areas. After liftoff pattering, rapid thermal annealing (RTA) was performed at 500 °C for 2 min to form an ohmic contact between Si and metals. The current (I)–voltage (V) characteristics were measured for each bonding pad to confirm ohmic contact. When the input voltage was applied from −1 V to 1 V, the current increased linearly in proportion to the voltage, showing that an ohmic contact was formed between metals and Si. After photolithography using a positive photoresist, 10 μm thick Si piezoresistors were patterned using a reactive-ion etching (RIE) process (Figure 7e). A layer of SiO_2_/SiN (50 nm/200 nm) was blanket-deposited over the whole wafer to passivate the strain gauges, then etched with RIE to open the Al wire bonding pads (Figure 7f). Finally, the lower glass substrate was polished so that the gauge die thickness was 50 μm (Figure 7g), and the dies were separated from the processed wafer by mechanical sawing, as shown in Figure 7h.

**Figure 8:**
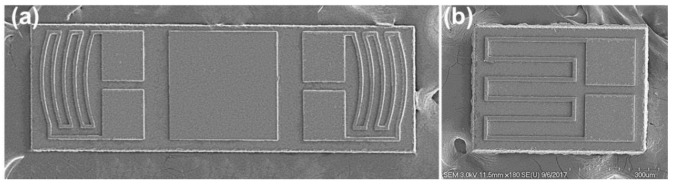
SEM images of the fabricated strain gauges: (**a**) dual-arc strain gauge (two tangential gauges) and (**b**) single linear (radial) gauge.

Figure 8 shows scanning electron microscopy (SEM) images of the fabricated arc-shaped and linear strain gauges. The gauge thickness was 10 μm, and each piezoresistor was 15 μm wide and 280~300 μm in length.

**Figure 9:**
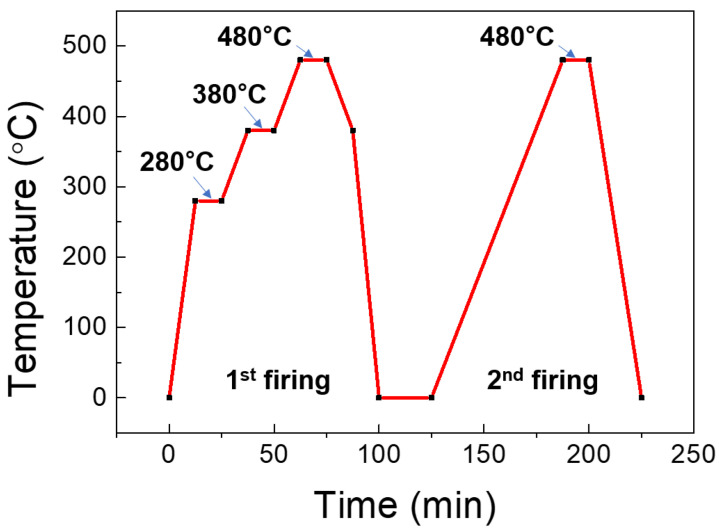
Temperature profile of the glass frit firing process.

**Figure 10:**
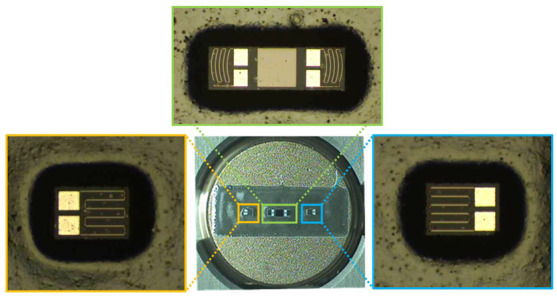
Images of the silicon strain gauges after glass frit bonding.

One arc-shaped gauge chip and two linear gauge chips were attached to the steel diaphragm using a glass frit to evaluate their properties and the feasibility of using these sensing elements to measure high pressure. Although several organic epoxies could also have been used instead of glass frit to attach the silicone gauge to the metal diaphragm, they negatively affected the mechanical and electrical properties of the sensor, such as creep, hysteresis, nonlinearity, reproducibility, long-term reliability, and operating temperature range [25,26]. The glass frit bonding process consisted of three steps: glass frit screen printing, initial dry, and first and second firing. First, the glass frit was screen-printed on the cleaned metal diaphragm surface where the device chip was placed. Second, the organic solvent was removed by drying at 120 °C for 30 min. Next, the first firing was performed according to the temperature profile as shown in Figure 9. In the process of raising the temperature in the dry and first firings, the solvent and the organic binder were burned-out. This process was essential because it prevented voids caused by organic residues inside the bonded glass, which could degrade bonding adhesion and reliability. Finally, the gauge chips were loaded onto the glazed glass frit and subjected to a second firing with a temperature profile similar to that of the first firing. Figure 10 shows images taken after glass frit bonding was completed.

## 4. Results and Discussion

Strain gauge sensors bonded on a steel diaphragm were attached to a high-pressure test manifold and placed in a temperature-controlled chamber during all tests, providing electrical connection to the test device. Each strain gauge was tested over five runs under specified conditions of time, temperature, and pressure. The resistance changes of strain gauges were measured with digital multimeters (3458A, Agilent Inc., Santa Clara, CA, USA) and recorded simultaneously in real time. For the full test, the excitation voltage remained constant at 5 V.

### 4.1. Strain Analysis

**Figure 11:**
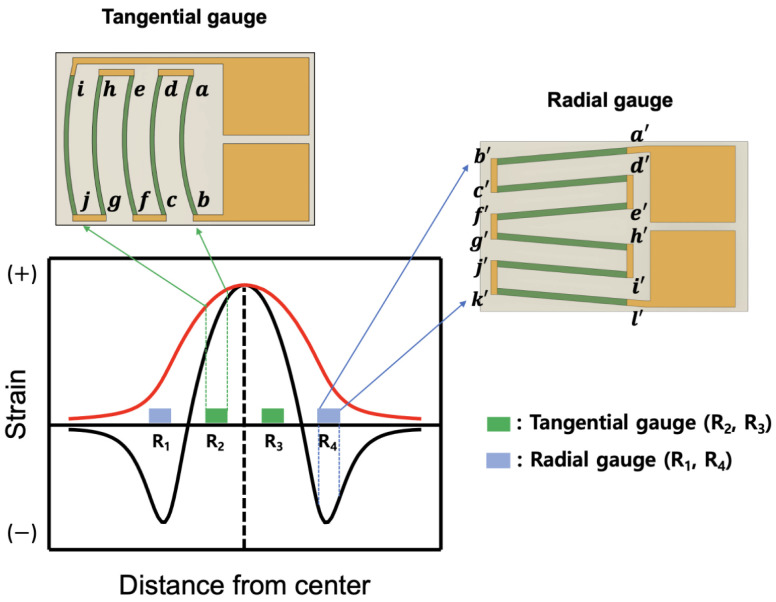
Simulated strain distribution and gauge locations on a stainless-steel circular diaphragm.

Figure 11 shows the induced stress at the locations of the tangential and radial strain gauges, showing a non-uniform distribution. The strain gauge tended to integrate or average the strain over the area covered by the piezoresistor [8]. Therefore, the strain gauge measured the average strain under the gauge grid. For the arc-shaped gauge, the strain along the piezoresistors was constant, but each grid responded to different average strains because the tangential strain varied along the radial direction. Therefore, the average strain over the area covered by the tangential piezoresistor can be calculated as

(5) $$\varepsilon_{average\left(tangential\right)\;}=\frac{\varepsilon_{ab}+\varepsilon_{cd}+\varepsilon_{ef}+\varepsilon_{gh}+\varepsilon_{ij}}{5}$$

where $\varepsilon_{ab}$, $\varepsilon_{cd}$, $\varepsilon_{ef}$, $\varepsilon_{gh}$, and $\varepsilon_{ij}$ are the average strains of the piezoresistors.

On the other hand, the strain along the radial gauge was linear, and the change in resistance was due to the average strain along the grid. Accordingly, $\varepsilon_{a^{\prime }b^{\prime }}=\varepsilon_{c^{\prime }d^{\prime }}=\varepsilon_{e^{\prime }f^{\prime }}=\varepsilon_{g^{\prime }h^{\prime }}=\varepsilon_{i^{\prime }j^{\prime }}=\varepsilon_{k^{\prime }l^{\prime }}=\varepsilon_{ag}$, and the average strain under the radial gauge was calculated as:

(6) $$\varepsilon_{average\left(radial\right)\;}=\frac{\varepsilon_{a^{\prime }b^{\prime }}+\varepsilon_{c^{\prime }d^{\prime }}+\varepsilon_{e^{\prime }f^{\prime }}+\varepsilon_{g^{\prime }h^{\prime }}+\varepsilon_{i^{\prime }j^{\prime }}+\varepsilon_{k^{\prime }l^{\prime }}}{6}=\varepsilon_{ag}$$

**Figure 12:**
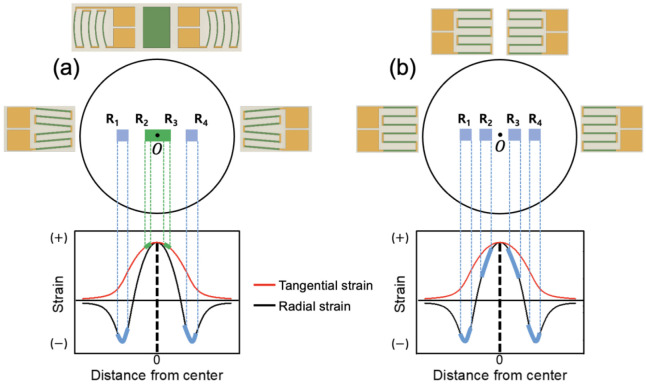
Installation positions of strain gauges on the circular diaphragm in two types of bridge configurations. (**a**) Full-bridge configuration consisting of newly designed three-gauge chips; (**b**) conventional bridge consisting of four single-gauge chips.

One of the most important problems in the design of diaphragm-type sensors is to determine the locations of the strain gauges composed of the bridge because the electrical output signal from the bridge is directly proportional to the net unit change in the resistance of all four arms. To achieve optimum sensor performance, a bridge with four active gauges subjected to equal and opposite tensile and compressive strains was required. Because the exact strain distribution and gradient on the diaphragm were known during the sensor design stage, the position of the measuring grid could be optimized. Figure 12 shows strain gauge bridge configurations discussed in this paper. In Figure 12a, one tangential gauge chip with two reciprocating arc-shaped gauges ($R_{2}\;\mathrm{and}\;R_{3}$) was placed around the center, and two radial gauges ($R_{1}\;\mathrm{and}\;R_{4}$) were placed near the periphery. Here, the strain gauges were installed in the area of high tensile and compressive strains to obtain the highest possible output voltage. For comparison, Figure 12b shows four single gauges, the most popular full-bridge configuration to date, installed in an area with the same tensile and compressive strains as shown in Figure 12a. Two tangential gauges ($R_{2}\;\mathrm{and}\;R_{3}$) and two radial gauges ($R_{1}\;\mathrm{and}\;R_{4}$) were bonded in symmetric positions as shown in Figure 12a,b.

The average strains under the gauges according to Equations (5) and (6) are listed in Table 1 for two full-bridge configurations. The conventional four-chip bridge in Figure 12b was added to this table for comparison purposes only. Because gauges R_2_ and R_3_ in the three-chip design responded to the tangential strain, the average strains acting on them were greater than the corresponding gauges in the four-chip design, in which all four gauges were subjected to radial strain. Therefore, the output of the three-chip full bridge in Figure 12a was expected to be larger than that of the conventional four-chip bridge.

### 4.2. Individual Gauge Response

**Figure 13:**
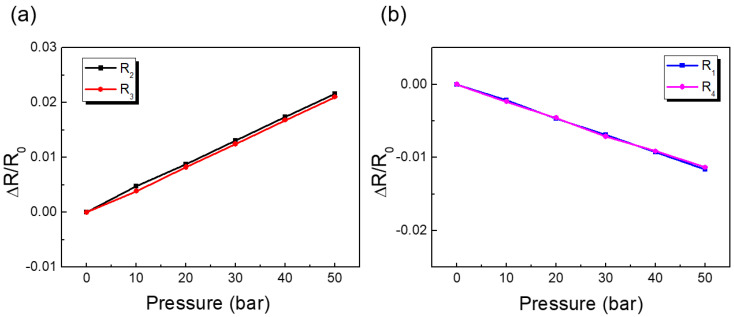
Variations of relative charge in gauge resistance with strain for (**a**) two tangential gauges and (**b**) two radial gauges.

Figure 13 shows the variation of relative change in resistance ∆R/R with strain for tangential gauges (*R*_2_ and *R*_3_) located near the center of the diaphragm and radial gauges (*R*_1_ and *R*_4_) located near the edge. As shown in Figure 13, the rate of change of tangential gauge resistance increased linearly with tensile strain, whereas the radial gauges showed a linear decrease in resistance with compressive strain. The input signal of a strain gauge was the strain (ε) to be measured while the output signal was the relative change in resistance (dR/R) generated by the strain (ε). The sensitivity or the gauge factor (*K*) of a strain gauge is defined as

(7) $$\frac{dR}{R}=K\varepsilon$$

The gauge factor of each sample was calculated using Equation (7) from the slope of curves shown in Figure 13 and listed in Table 2. It was observed that for all the gauges, the variation in ΔR/R with strain was linear and repeatable (between gauges). This is excellent performance, given that the gauges were selected randomly from the processed silicon wafer.

### 4.3. Characterization of Full-Bridge Output Using Pressure

In practice, as the diaphragm was stressed by pressure, resulting in small strain, as shown in Figure 6, and causing a very small change in the resistivity of the gauge. As a result, four strain gauges are nearly always coupled in a Wheatstone bridge configuration to record minute changes in resistance related to strain and adjust for temperature sensitivity. As shown in Figure 13, a positive tensile strain occurred on gauges R_2_ and R_3_, and a negative strain was experienced by gauges R_1_ and R_4_. The bridge output was proportional to the sum of all the strains measured separately and was found to be four times the output of the quarter bridge.

**Figure 14:**
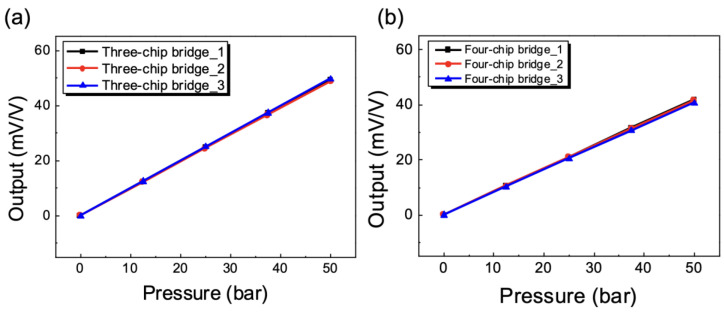
Output voltage vs. pressure curves of three- and four-full-bridge circuits: (**a**) three-chip bridges; (**b**) four-chip bridges.

Figure 14 shows the output results for the three-chip and four-chip bridges with applied input pressures from 0 to 50 bar. The variations in output voltage as a function of applied pressure were linear and parallel. The net output, when the bridge offset was eliminated, showed negligibly small span shift, as shown in Figure 14. Sensitivity was calculated from the slope of an end-point straight line for each bridge.

(8) $$Sensitivity\left(mV/V/bar\right)=\frac{Voltage\;Output\;\left(mV\right)}{Supply\;Voltage\;\left(V\right)\times Pressure\;Input\;\left(bar\right)}$$

Table 3 summarizes the main output characteristics for the three-chip bridges and the four-chip bridges in Figure 14. The average sensitivity of the three-chip bridges with an arc gauge and four-chip bridges with a single gauge was about 0.991 mV/V/bar and 0.831 mV/V/bar, respectively. The sensitivity of the three-chip bridge is about 1.2 times that of the conventional four-single-gauge bridge (Figure 14b). The maximum nonlinearity, defined as the percentage deviation of the calibration curve from the best-fit straight line, was recorded as 0.99 %FS. Hysteresis error, which is the maximum deviation between the increasing and decreasing characteristic curves at a specified point in the input pressure, was found to be 0.005 %FS.

### 4.4. Temperature Effects on Output Characteristics

An important characteristic of a silicon strain gauge is its temperature stability. In reality, every pressure sensor’s output is slightly affected by several environmental variations and fluid temperature. Changes in temperature not only affected the gauge resistance but could also cause the sensing materials, pressure medium, and housing to expand and contract [27]. These factors influence how the resistance and sensitivity (gauge factor) of a gauge change with temperature. The temperature coefficient of resistance (TCR) of the bonded silicon gauge is the combination of the TCR of the silicon gauge plus the differential thermal expansion between the silicon gauge and materials to which the gauges were bonded. Typically, the behavior of a sensor in terms of changes in temperature is characterized by two temperature coefficients: a temperature coefficient of the offset voltage (TCO) and a temperature coefficient of the span (TCS).

**Figure 15:**
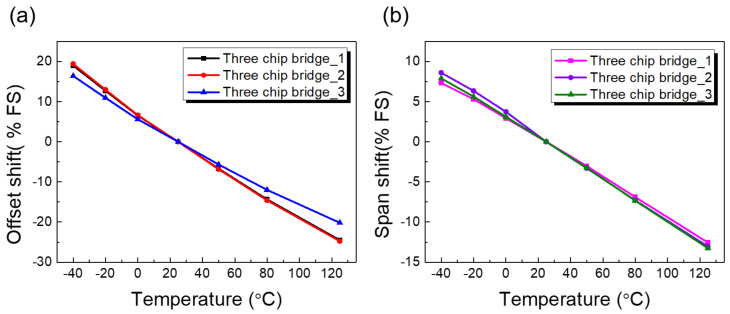
(**a**) Offset drift and (**b**) span drift results of a strain gauge with temperature variation.

Figure 15a shows the curve for the offset shift. The offset TC is not usually a straight line. The curves have a slight bow depending on how closely the offset TC matches the compensation circuit. Because a number of factors could affect the offset, each sensor must be tested under varying temperatures to determine the sign and magnitude of the compensation. The sensitivity of silicon gauges decreased linearly with increasing temperature, as shown in Figure 15b. This linear dependence on operating temperature range allows end users to compensate for offset and span shift temperature with a very simple algorithm. Any deviations in the linear function, including those occurring over time and as a result of exposure to environmental conditions, manifested themselves as sensor errors. The temperature coefficients of offset and span were evaluated as 0.22 %FSO/°C and 0.10 %FSO/°C, respectively.

### 4.5. Long-term Stability Test

Factors such as thermal and mechanical stress can negatively affect the long-term stability of pressure sensors. However, these effects can be minimized through diligent testing during production. The long-term stability of pressure sensors is usually determined under laboratory conditions, and it refers to the expected maximum change of zero point and output span. In order to ensure long-term reliability, sensors were tested for shifts in offset and span resulting from thermal stress conditions.

**Figure 16:**
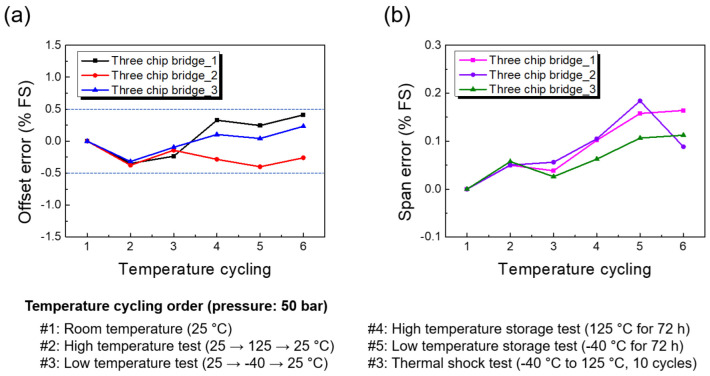
Variations in offset (**a**) and span (**b**) errors after temperature cycle/shock tests.

Figure 16 shows the variations in TCO and TCS after a thermal cycle and shock test. After the low-temperature cycle test, the offset curves were separated, indicating that the differences between three devices had increased, but with different offset errors than before. In general, more attention should be paid to the zero-point shift, as the stability of the pressure sensor mainly depends on the offset voltage, and TCO is easily recognized. Unlike thermal zero shift, TCS is a systematic error and is much more predictable than TCO, which is a random error, as shown in Figure 16a. Practice showed that the new pressure sensors usually take some time to stabilize. Therefore, they should be aged and subjected to thermal tests before leaving production.

## 5. Conclusions

In this paper, we present a new design and manufacturing approach for silicon strain gauges used in metal diaphragm pressure sensors. The novelty of this work is to reduce the number of gauge chips attached to the diaphragm while increasing the output sensitivity of the bridge. A tangential gauge chip consisting of two reciprocating arc-shaped strain gauges (R_2_ and R_3_) was designed and fabricated on a silicon-on-glass (SOG) substrate using a MEMS-based Si process. The two radial gauges near the periphery were in the form of a meander with series-connected linear piezoresistors responding to compressive strain. In a three-chip design, two arc gauges (R_2_ and R_3_) measured tangential strain, which was much larger than the radial strain, while the conventional bridge used four single gauges responding to radial strain. The results show that the full bridge with arc gauges has much higher output sensitivity than the conventional four-single-gauge bridge.

Finally, it is important to note that the design approach proposed in this paper used thin glass for the gauge backing, which greatly improved the breakdown voltage and made it easier to handle in the bonding process. Arc gauges can also be extended into half-bridge and full-bridge configurations to further reduce the number of glass frit and Al wire bonds in the diaphragm pressure sensor and to achieve higher sensitivity by reducing the thickness of the strain gauge.

## Figures and Tables

**Table 1:** Average strain for three-chip and four-chip designs.

Bridge Configuration	Average Strain (mm/mm)			
	R_1_	R_2_	R_3_	R_4_
Three-chip design	$-1.574\times 10^{-4}$	$1.928\times 10^{-4}$	$1.927\times 10^{-4}$	$-1.579\times 10^{-4}$
Four-chip design	$-1.533\times 10^{-4}$	$-1.597\times 10^{-4}$	$-1.596\times 10^{-4}$	$-1.538\times 10^{-4}$

**Table 2:** Summary of the sensitivity of tangential and radial gauges according to Equation (3).

Strain Gauge	Gauge Factor (K)
R_1_ (Radial gauge)	103.54
R_2_ (Tangential gauge)	104.27
R_3_ (Tangential gauge)	105.37
R_4_ (Radial gauge)	101.97

**Table 3:** Comparison of output characteristics of a three-chip bridge and a four-chip bridge.

Full Bridge	Sensitivity (mV/V)	Linearity (%FS)	Hysteresis (%FS)
Three-chip bridge (Figure 14a)	0.991	0.999	0.005
Four-chip bridge (Figure 14b)	0.831	0.999	0.004

## Data Availability

The data presented in this study are available on request from the corresponding author.
